# Sir George Lefevre on the Pathology of the Nerves and Nervous Maladies

**Published:** 1849-01-01

**Authors:** 


ART. VI.—*An Apology for the Nerves, or their influence and importance in Health and Disease.* By Sir GEORGE LEFEVRE, M.D. Longman and Co.

THE dominion of the nerves over the mental and physical actions of man is unbounded. The various workings of intellect, sensation, volition, motion, secretion, nutrition,—the essence of all these is nervous influence.

With this admission, we approach the author of this "Apology," as it is termed, "for the Nerves," adopted, we presume in regard to the distressing maladies which the varied degrees of sensibility entail on human nature. The eligibility of this title we will not here dispute, although some may conceive that the apology should rather emanate from ourselves.

We cannot, in limine, pin our faith on the author, regarding his affirmation of the paramount influence of the nerves. This is the triumphant motto adopted, from Maculloch, by Sir George Lefevre—

> "There is no animal without nerve, but myriads without the circulation of blood."

Now this sentence implies nothing more than that where there is no circulation it is not wanted, for, in beings thus fashioned, the Creator has ordained that the physiology of nerve should be obedient to different laws. We might justify this difference, by asserting that there are vegetables which secrete, but have no nerves: for the most bigoted and romantic Darwinian will not, we opine, identify the contractions of the Mimosa, or the Dionæa, with the vis nervosa (myotility, as Elliotson terms it) of Haller, or the last flickering of contraction on the electro-stimulation of a dead muscle. We are not, however, quite so bigoted to the dogmas of the humoral pathology, as to refer all disease to vitiated blood: we wish merely to vindicate the equal importance of blood and nerve in regard to the phenomena of health and disease.

Granting, then, that *primary* impressions often fall on the nerve—the secondary effect being their influence over the circulation: still it must be remembered, that it is this very secondary condition which is so prominent in pathological interest—constituting in itself, or its products, what we term the proximate cause of disease.

Thus, alarm or terror are mental impressions: but how instantaneously the heart is affected by undue enervation is evidenced by palpitation, increase or arrest of circulation, by blush or pallor.

There cannot, of course, be any atom of the vascular tissues of the body, the integrity of which does not require an adequate supply of healthy blood. The phenomena of mental disorders, or of a class of them at least, are dependant on the disordered conditions of the crasis of the blood, perhaps, we may say, on an abnormal supply of carbonized or oxygenized blood on the brain, or a deficiency of phosphorus.

The nerves, even to their ultimate fibrillæ, come under the same category: congestion, or a remora of venous blood, equally inducing an impediment to *their* function, as to that of the cerebrum. Excess of arterial or venous blood will equally induce a train of morbid phenomena in the nerve, as in any other organic tissues of the body.

Blood-pressure on a nerve will produce sensation of a most painful nature.

Thus Bichât—

"I preserve the sciatic nerve of a subject, who suffered very acute pain in its course, and which presents, at its superior part, a number of small varicose dilatations of veins penetrating it."

And Swan—

"In examining the limb of an old man, who had died of mortification of the foot, after a very gradual and very painful extension of the disease from the toe, I found the sciatic nerve loaded with veins containing blood; the arteries of the whole limb were very much ossified. In those cases of painful affections of the nerves in which the limb is cold, and the pain is prevented or relieved by warmth, I conceive the languor of the arterial circulation may favour the congestion of the veins of the nerves, and thus produce distention of their fibrils; and that warmth, by increasing the action of the arteries, and favouring the return of the blood, and the cutaneous exhalation, may take off or prevent this loaded state of the veins, and relieve the nerves."

We may see daily, how obstruction to the circulation of a nerve will be instantly and invariably followed by imperfection in the function of the part:

"In some cases, a sudden and irregular action of the bloodvessels of a nerve may cause tic douloureux, in the same manner as an increased and irregular action of the vessels of the choroid coat produces dizziness: that the bloodvessels have a principal share in the production of pain in some cases, may be inferred from their increased size in nerves that have long been thus affected. In the musculo-cutaneous nerve, Mr. Hunter was obliged to use a ligature to stop the hæmorrhage, which could never have been necessary in a sound nerve. In another case, the peroneal nerve bled very profusely, and the portion removed was much redder than a sound nerve."

"If the nerve in tic douloureux be affected by its bloodvessels, in the same manner as the retina is by the choroid coat in dizziness, tic douloureux ought to arise from a too plethoric state of the body, as well as from a debilitated one; and this in fact is the case. It may appear strange, that a complaint of this sort should be produced by two such opposite states of the body, and that it should continue longer in a debilitated person than a plethoric one: but when the body is strong, the nervous system is not irritable: slight occurrences do not easily affect it, so as to produce any disturbance in the sanguiferous system: whilst, on the contrary, in the atonic state of the body, the brain and nervous system are generally very irritable, and the action of the heart is easily hurried; and this susceptibility tends to increase the irritability of the brain and nerves, by causing the blood to flow to them at irregular times.

"Although the irritation of the nerve be the cause of the increased action of the bloodvessels, yet this may tend to increase or keep up the irritation of the nerve, and even to continue the local disease after the cause has ceased to exist."

We have advanced thus much to assert, with more than our own authority, the equal importance of the vascular and nervous systems quoad their influence on health and disease; and to strengthen our point have quoted an author, whose very province is the physiology and pathology of nerve.

Dr. Lefevre affirms, very truly, that the division of two small cords will stop respiration: so let the coats of a minute capillary be torn, and life is gone in an instant. So, if Sir George adduce that species of sudden death, Nervenschlag, which is marked, he allows, by "considerable turgescence in the vessels of the brain"—we may refer to apoplexy as its prototype. *Extravasation* is not essential to apoplexy. If vitality itself depend on due innervation, to insure this there must be a due supply of blood to the nerve.

*We* do not, however, offer an *apology* for thus implicating the blood in the production of disease—of course, we need none for its physiological quality—this would imply a *fault* in our organic creation. If there must be apology, ours will rather be for freely commenting on the work of a learned author, who has ceased to be agitated by brain or heart, having passed to that state in which there is being without the influence of nerve or blood.

The discursive nature of Sir George Lefevre's book is somewhat puzzling; it is, indeed, a farrago in one sense, and an unhappy example of that mercantile spirit for the manufacture of a book, by adding make-weights at the end, quite irrelevant to the title-page. We all remember the successful scheme for selling off an edition of "Drelincourt on Death," by tacking on the imposture of Mrs. Veal's Ghost, as a sort of appendix, just as a lady's letter contains the cream of the correspondence in the postscript.

In our comments on this book (in which we had hoped for *that* redeeming quality), we must proceed, with a sort of running fire, not confining ourselves to a regular critique, but taking up Sir George Lefevre's points—i. e., such points as touch the wording of the title-page, just as it may suit us.

There has been much critical ink shed in the cavil, whether the principle of life be in the blood or in the nerve; but we have not yet demonstrated the physiology of the Hebrew lawgiver—"the blood is the life thereof"—unless we adduce the negative assumption, from the experiments of Wilson Philip and Legallois, to prove that life may exist without a nerve, by arresting the process of one of its functions, digestion—by the division of the eighth pair, and renewing it by the influence of galvanism.

If, however, we lean to the contrasted hypotheses of Abernethy and Lawrence, we might, from different conclusions, decide that the vital principle does not exist in either.

It has been demonstrated that bloodless muscle will contract under the influence of galvanism; but this is but the evidence of a lingering scintillula of that irritability imparted during life, by the combined agency of nerve and blood, to the stomach, which ceases to digest if the eighth pair be divided. Galvanism will renew the function; but mere innervation would not effect this—it is by the secretion of an acid from the blood, the secondary process. So *if*, in suspended animation, scarlet blood can be introduced, by any mode, to the left side of the heart, vitality may be restored. The heart obeys its stimulus, and obeys it equally as if the galvanic aura be transmitted through it.

Now, without presuming to decide that the principle of life is equally dependent on nerve and blood, we cannot but believe that their influence on health and disease is combined and mutual. There is, moreover, some specific quality in the blood of different classes of living beings; the herbivora will not be sustained by the blood of the carnivora; nor can man be revived by the transfusion of the blood of a brute.

Now, in syncope from hæmorrhage, Sir George Lefevre says the brain is deprived of its nervous energy; but the nerve itself is exhausted—how? by the hæmorrhage: its blood is withdrawn, so both systems are in a fix—at a "dead lock," as Sheridan has it. In one sentence: blood does not stimulate nerve, and nerve, in revenge, stops the current of blood. So there is this mutual dependence in physiology: if the gland will not secrete without its due innervation—so, first, the nervous energy must be imparted—we will presume to write, the *nervous fluid* must be secreted from the blood.

We cannot demonstrate at what period of evolution vitality is imparted to newly-formed tissue; whether the first molecule set in action be nerve or vessel: but the last *act* of life is hæmal—the coagulation of the blood. And we even infer the importance of the circulation in fœtal development from the fact, that the primal deposit of neurine is the *cardiac* ganglia.

The quality which we term idiosyncrasy—that peculiar susceptibility to impression, or tendency to change, which we term constitution—may superficially be referred to the condition or state of nerve. But the power of resistance to disease is a combination of nervous and vascular energy. It is true, we have many very interesting recitals of the power of mind in fortifying the body—such is the prophylaxis of courage. The story of the Governor of Marseilles—his immunity during the raging of the plague in that city, while his timorous soldiers sunk under the pestilence, is too hackneyed a tale to need repetition.

But the essence—the proximate cause—is it not vigorous circulation? At any rate, whatever induces a free and healthy circulation of oxygenized blood, as exercise, previous to the exposure to infection, may entirely ward off the evil. By analogy we know, while we are riding over the Pontine Marshes, how essential it is, not only to close the carriage-windows, but to keep the body and the mind awake, for if we sleep we catch an ague. And why? During the slumber, the circulation becomes slower, and the power of resistance is weakened. So, if two persons—the one vigorous, the other languid—are exposed, the one may escape—the other is almost certainly affected.

So, the higher the nervous energy, or the more vigorous the circulation, the greater the power of the vital tissues to resist putrefaction. If the body be dead, deprived of both these actions, we know it is speedily decomposed.

Hope will preserve the energy of the body under the most depressing physical influences—the most laborious exertions. Hope, however, is not only felt sensibly in the heart, but it almost ensures a vigorous circulation. On the contrary, it is known how the circulation of the soldier is affected so soon as an army commences an inglorious retreat; the pulse becomes irritable—languid; the respiration slower, and irregular—the apathy, or asthenia of disappointment. In the benignant influence of hope and buoyant spirits on the blood, consist the most magical effects of change of air. To a certain degree, even a series of deep sighs will effect a similar change. On a full inspiration, a flush or glow steals over the frame; we feel happier, and healthier thoughts are usually the consequence.

It is in the stimulus of confidence and faith that the influence of Mesmerism—the virtues of the amulet or charm, and the miracles of Hohenlohe, consist. We know a gentleman who is always relieved of subacute tonsillitis, by wearing a skein of black thread around his neck: is the virtue in the thread or in the patient's faith. Thus, even without a divine miracle, we may be assured that faith may often make us whole. To this influence is also allied the stimulus of necessity: there is a report of the extreme facility with which a body of soldiers stormed a fort; but when they were subsequently commanded to repeat the attack, during a sham fight, not one could succeed in mounting the breach.

We follow Sir George Lefevre in his glances at the influence of the nerve in paralysis, for the purpose of expressing our belief that, when this state of defect is induced by pressure on, or division of, a nerve, it more speedily suffers in its evolution of caloric, the animal heat rapidly sinking below 94° of Fahrenheit—the degree of temperature in health. We will not pause to discuss the point of difference between Dr. Elliotson and Sir George Lefevre, as to this evolution being a chemical or a vital action, but at once refer to congestion as a very common source of paralysis, its termination being, if unchecked, the loss of vitality—gangrene. And what is the remedy?—not the galvanic aura, but friction, to restore circulation. This is so well known in Boreal climates, that a stranger will very unceremoniously rub the nose of another, whom he casually meets, with snow (as in the instance alluded to of the postilion of St. Petersburg), his only apology being the salvation of that important feature in his patient's physiognomy. We will, to strengthen this point, quote a few words of Dr. Marshall Hall:

> "There is, indeed, a marvellous connexion between the nervous and vascular systems throughout the animal frame. Too great action in the minute arteries, congestion in the veins, an anæmious state of the vascular system of the encephalon, alike induce morbidly-exalted and impaired conditions of the mental and cerebral functions: spectra, delirium, insomnia; amaurosis, stupor, coma; violent voluntary actions, or paralysis of the voluntary motions, &c."—*Diseases and Derangements of the Nervous System.*

To carry on his eulogy of nerve, Sir George Lefevre comments on the paramount protection of the cranium; but there is equal preservation of the heart from common injury, the elastic sides of the thoracic cavity being as completely preservative as the unyielding cranium. We do not admire these maudlin eulogies of *one* of God's works above another. Every atom of creation indicates the perfection of design in the Creator. It did not need the bequest of the eccentric Earl, of £10,000, to prove this; although we confess a very fair apology is offered, in the acknowledged excellence of the Bridgewater Treatises.

One of our most interesting subjects is the remote influence of moral causes on corporeal functions. The effects of emotion are daily witnessed, but are not sufficiently studied. Some of these are strictly nervous influences, and they are too rapid for us to believe that the circulation has had time palpably to change its state. *After awhile*, however, this change *will* ensue; for not even a thought, we believe, passes through the brain without action, and a relative change in the condition of tissue. An abstract passive influence is a paradox; there is no such thing as mere metaphysical pathology.

Even a very remote nerve may be influenced by local irritation, without any evidence of this in the nervous tract which is traversed; just as the electric fluid may pass unperceived or unfelt through a wire and evince its power by the discharge of the jar. If we merely touch ourselves in one part, we frequently feel a sensation, perhaps, in the most distant point of the body. And here Sir George Lefevre has overshot, we think, our own mark, in observing that "we cannot get to an individual nerve but through the circulation."

There are still more impressive phenomena that very beautifully illustrate the remote influence of nerve in the potency of volition. The power of fixing the thought, or concentrating the will, is sometimes intense—we may almost term it miraculous. Thus Colonel Townsend possessed the wondrous faculty of arresting the action of his heart, which feat, indeed, at length, terminated in unintentional suicide. Almost equal power was possessed by Coma, as we learn from Valerius Maximus, who actually committed self-murder by the unique faculty he displayed, of stopping his breathing by the effort of his will.

We have seen very curious instances of the power which some possess, of influencing function, by the fixing of attention to the point. We may, indeed, often induce the bowels to act, by thinking of their action; as we may also in reference to the emptying of the bladder; and this especially in a state of solitude. If the mind be distracted with the avocations of business, the call ceases, even if it has ever been excited; the disposition not recurring until the usual hour of the next morning.

During anxiety, and while the circulation of blood is in excess on the brain, equally by profuse sweating and the draining off of the fluids of the body, the bowels become apathetic and disobedient, constipation thence ensuing. The converse of this: there are states of the circulation, those associated with pleasurable or buoyant thought and feeling, or a favourite subject of study, that in some almost invariably induce peristaltic action. It is the free circulation of oxygenized blood that sets the healthy functions a-going.

In illustration of this we may adduce the following fact: the contemplation of a map, by a gentleman ardently attached to the study of topography, was speedily followed by intestinal action, especially if the map was that of a country celebrated for beauty or local interest.

In illustration of the effect of mental anxiety, or concentration of attention, Dr. Lefevre tells us of a gentleman who was forewarned by a homœopathist that he would spit blood on such a day: he was really affected suddenly with hæmoptysis. But was this more than coincidence, or, at most, the secret of the fulfilment of prophecy, from *imparted impetus,* so often referred to supernatural agency? If one in a million of warnings is fulfilled, of course it is vaunted as a prophecy. "We mark," writes Lord Bacon, "what we hit, not what we miss."

Some of the *inverted* organic actions, however, are in some persons very suddenly induced by intensity of thought or emotion. A gentleman some years ago consulted us, whose stomach was instantly excited to eject its contents, whenever, on adjourning from the breakfast-table to his counting-house, he opened a letter containing disastrous or even merely unfavourable news.

From the same cause engorgement of the liver, or spasm of the biliary ducts, may also arise. When Murat, while he was in Russia, received bad news from Naples, he was very quickly affected by a severe fit of jaundice.

We believe that in many of these *emotional* influences, a revulsion of blood to the heart may ensue—the brain being anæmiated. In sea-sickness, moral influence may be brought to bear with much advantage. If the mind can be induced to indulge in, or turn to thoughts of a happy or absorbing nature, the convulsive actions of the stomach may be thwarted. By this effort blood is sent in due quantity to the brain, as it is in the recumbent posture. We ourselves were witness of this power during a late very tempestuous voyage from France. In the saloon were ten gentlemen, of whom eight were very seriously indisposed. One of those who escaped kept under the qualms, which threatened once or twice to gain the mastery over him, by concentrating his reflections on the beautiful works of art he had been deeply contemplating the day before. The subject of sea-sickness is one of more than common interest, but we must waive its discussion here—merely observing, that it might well be classed among the neuroses of Cullen. The influence of thought on secretion and digestion is chiefly dependent on varied degrees of anæmia: in that organ in which the paramount action is then going on, there we should find hyperæmia, whether this action consist in increase of function, or physical excitement.

The mucous membrane of the stomach and intestines, especially where sudden death, by disease or violence, has occurred, will be often seen of a bright or a deep scarlet hue—a condition which Haller and other pathologists erroneously believed to be the indication of inflammatory action, but which may, indeed, be deemed an essential state for the due function of those organs. When, however, we study deeply, directly after feeding, the brain is the organ in which nervous and vascular action is, as it were, concentrated. Digestion is impeded, not by deep thought, *metaphysically,* not by mere enervation, but by defect of its supply of blood. There is, in fact, a sort of struggle for the blood between the brain and the stomach, during which both play badly. Why do we not obey nature in this?—for she does indeed *indispose* us for study after a meal, but if we will force or oppose her, we must take the consequences.

Some curious allusions are made, in the book under our notice, to the influence of accident—as concussion of the brain, for instance—in suddenly changing the disposition. As one may be reduced to a state of fatuity, another may also, by some wondrous change in the circulation, be for a time rendered rational. Something of this we sometimes witness in the lighting up of reason just before dissolution. Among others, Dr. Hancock records the case of a Quaker, who had been long a drivelling idiot: shortly before he died, he became so perfectly rational, that he called together his family, and bestowed on them, with pathetic solemnity, his parting benediction. This illumination of the mind, this transient beam of reason, is finely employed by Mrs. Opie, in her tale of the "Father and Daughter."

Aretæus accounts for this by asserting that "The system has thrown off many of its impurities, and the soul, left naked, is free to exercise such energies as it still possessed."

This impurity was probably some morbid state or oppression of blood. Thus, timely venesection might have prevented many a suicidal crime. We could refer to more cases than that of the illustrious statesman, who had *rationally* attempted to stanch the life-blood flowing from the wound he had inflicted in his neck. Dr. Marshall Hall records one of attempted suicide, in which the flow of blood directly changed the nature of thought—the disposition, as Sir George Lefevre would write. The observation which a person who committed suicide made to his surgeon, as he was recovering from a state of syncope, was striking: "Had you bled me a few days ago, I should not have done this act; my feelings are altered; I regard suicide with abhorrence." An analogous case is recorded in the late work of Dr. C. M. Burnett.

We come now, somewhat suddenly, on sleep and dreaming; and we confess our disappointment at seeing so little discussion on these deeply interesting phenomena; but perhaps so wide and hackneyed a field may have deterred Sir George Lefevre, who might think, as we do, that a very certain mode of inducing sleep in ourselves, would be to peruse the myriads of hypotheses, and hard words and definitions, contained in the writings of psychologists. Just for a few of these :—The exhausted irritability of Darwin, the diminished afflux of blood of Blumenbach, the lack of animal spirits of Haller, the cerebral collapse of Cullen, congestion of sinuses, reflux to the heart, a deposition of fresh matter on the brain, &c. &c. We have elsewhere discussed this interesting subject, so beautifully illustrative of the mutual influence of nerve and blood.

In quoting Sir Astley Cooper's case, in which pressure within a hole in the cranium, made by the trephine, induced *sleep*, we cannot agree with the term. The state thus induced was that of coma, a *disorder*, which sleep is not—it is *the remedy for disorder*. One point only on this subject we will refer to—the power of slumber in quickly altering the sentiments or the complexion of thought. We have often experienced this happy influence of a very transient slumber ; and Sir George Lefevre knew a gentleman who was often disturbed by a confusion of ideas, which were constantly renovated or rectified by five or ten minutes' sleep. The exaltation of one sense, when vicarious of another impaired or lost, may almost realise the clairvoyance of the mesmerist. Practice and habit may do much, but something must be referred to the excess of that nervous and vascular energy once expended on the lost sense being superadded to the substitute.

The exquisite acuteness of touch in Professor Sanderson, of Dr. Blacklock, and Miss M'Avoy (written Macaulay by Sir George Lefevre), are well known. In Laura Bridgman, of Boston, the whole faculty of *perception* was concentrated in touch. In some rare instances, conversation has been kept up by tracing letters on the *clothes of the back*, or on one side of the face, or by a whisper slightly breathed on the pit of the stomach of a deaf woman. In Caspar Hauser, in whom there was very slight working of intellect to carry off power, the sense of touch was intensely acute. How far Sir George Lefevre is correct in referring to stricture of the intestines, in two ladies, extreme exaltation of the senses of vision and hearing, we will not presume to determine.

Our author next refers to a curious case, in which a convulsive malady so changed the integrity of vision, that red and yellow things appeared green. In some this faulty discrimination of colour, if so it be, is *natural* and permanent. This arises from the different power, in different primitive colours, of the ray of light, of refrangibility, on which depends the interesting optical law of accidental or complementary colour, whether this be referred to the optical apparatus or nerve of the eye. If the eye be *strained* on a red colour, it becomes at length insensible to it, *perceiving* only the yellow and blue rays, the blending of which is green. This is easily illustrated by a red wafer in a bright light.

One of the most interesting among the nervous diseases of the eye, is "muscæ volitantes," whether this proceed from systemic sensibility or over-exertion of the sense. The spectra are not, however, always depending on mere nervous causes—sometimes on vascular turgescence. The one will be often relieved very quickly by a glass of wine, the other will require depletion. We have at present under our care a gentleman whose aspect is the picture of health, but myriads of these black floats are constantly before his eyes. We refer this disorder to his almost incessant employment, during one season, in looking up to the heads of forest trees for the purpose of valuation. Depletion is *always* followed by relief; as yet, however, this relief has been merely transient.

Of the antipathies of smell and taste we have known very curious instances. The olfactory nerves may become so acutely sensitive as to be oppressed by even grateful odours, so that Pope scarcely exaggerates in his lines—

> "And quick effluvia darting to the brain,
> Die of a rose, in aromatic pain."

The smell of strawberries, cheese, and malt liquors, is to some persons so nauseous, that we have known a gentleman of great energy on the verge of syncope during an *attempt* only to taste the beverage from one of Barclay's immense vessels, which others were eulogizing as the drink of the gods.

The Schneiderian membrane in some persons is endowed with peculiar sensitiveness to the vegetable odours. To this may chiefly be referred the disorder termed hay fever, but which is sometimes induced by the aroma of other flowers, as those of the blackthorn, &c.

The perversions of taste are sometimes very curious. We are informed of an old lady whose taste was *saline,* so that she did not require salt to her animal food.

Regarding the aid afforded by the nose to the palate, Sir George Lefevre writes, that we can taste but not *flavour* without our smell; so we hold a child's nose when we give it physic. But the palate and nose are not always so discriminative; the eye is sometimes called in aid. It has been often proved that mutton and beef roasted cannot be certainly distinguished if the eyes be shut.

The morbid eccentricities of touch or feeling are among the most painful maladies of the neuroses. They are eminently characteristic of hysteria. A lady, who was for several years under our care for phthisis, was occasionally affected with intense hyperæsthesia of the skin. During this state, a feather dropped on the abdomen would instantly produce such intense agony as to draw forth a shrill and prolonged scream.

In some women, the uterine sympathies during gestation will induce a state of hyperæsthesia or pruritus, that may banish sleep for several successive nights. A lady, on whose case we were consulted, in Sussex, was thus tortured, nor was she entirely relieved of the malady until her child was born. The urine in this case was albuminous; it is often so during parturition, without eliciting any complaint; but the liquor potassæ and digitalis seemed to produce so much amelioration, that I have been induced to think that the kidneys are often a sort of go-between; at least, they and the skin are constantly vicarious.

Among the local hysterical affections, as Sir Benjamin Brodie terms them, one of the most frequent is acute pain in the knee-joint. Indeed, the affection is marked by almost every sign of structural disease, save that it is more diffused. Yet this is true neuralgia; and Sir Benjamin Brodie concludes his sentence thus :—"I do not hesitate to declare, that among the higher classes of society, at least four-fifths of the female patients, who are commonly supposed to labour under diseases of the joints, labour under hysteria, and nothing else."

On the importance of the integrity of the cutaneous tissue, as a prophylactic against disorders, Sir George Lefevre lays much stress, and very justly so. The bath and flesh-brush, resorted to with judgment, would ward off many of the ills that flesh is heir to. The permanent benefit is from combined action, more probably that of inducing free transpiration, and restoring or conserving the healthy physiological quality of the depurative process, as well as that of reaction and counteraction. There is one curious fact in reference to sensation—the comparison of one portion of the cutaneous surface with another. There is a sort of *jealousy* of the flesh-brush (*risum teneatis amici?*), proving both its pleasurable and salutary influence. We shall find, although there has previously been no uneasiness in the skin, that when one portion of the surface has been well rubbed, that which *has not* directly *itches*, and remains uneasy till it also be rubbed. The disturbance of electric property, on which this probably depends, would form, elsewhere, an interesting topic.

Sir George Lefevre tells a very odd story of what he would term, we suppose, *antipathetic neuralgia*. A neuralgic lover became so absolutely disgusted with the mere touch of the hand of the lady to whom he was betrothed, that he could not be prevailed on to take her hand in his. This, however, is too quaint a tale to fall under the category of depraved sensation; it is nothing less than monomania, closely allied, perhaps, to that strange infatuation, by which the most devoted attachment is converted into the deepest hatred; an illusion frequently witnessed in sensitive young girls, in regard to their once dearly beloved parents, and we may add those mysterious cases of child murder, in which the infanticide has ever expressed the most ardent devotion to the child.

The neuroses of the vocal organs combine some of the most distressing though common maladies, especially in young females.

We have seen cases of complete loss of voice of many days' duration from the mere disturbance of innervation, coming on suddenly, and without the slightest structural disease.

To one young married lady we were more than once summoned, who in a moment *lost her voice,* being scarcely able to express her meaning in the lowest whisper. It was at the hour of midnight that we were usually called to this lady; and from this, as well as from the *confessions* which were made to us, we confidently refer the loss of voice to sexual sympathies.

The diffidence which sensitive girls experience in singing in public is closely allied to this aphonia. We knew one young lady, especially, who, in private or by herself, sang with perfect science and melody; but *before company,* her voice became hoarse and discordant, or sometimes scarcely audible.

It is so, often, with the young orator—one who might have prepared and learned by heart, in his study, the most elaborate address; but, lo! when he rises in the senate to repeat it, he can get no farther than—"Mr. Speaker, I am truly sensible—'hem!—ha!—" and there he stops.—Vox faucibus hæsit!

Now if these unhappy actors could but think only of the subject of their efforts, and become absorbed in the *sentiment,* the impediment would not occur. This concentration or abstraction, call it what we will, is the secret of those almost supernatural exhibitions of mesmerised girls. Mr. Headland related to us the case of a girl, which he and many others witnessed, in whom there was induced, by mesmeric passes, such an exaltation of power, such an intensity of melody in the voice, that her singing both astonished and delighted her audience—she herself being unconscious of her exalted energies. The intensity of this quality is that high qualification for stage acting—*abandonment.* No one could look on the gifted, though unhappy Malibran, during her exquisite *scena* in the "Sonnambula," without a sentiment almost amounting to adoration. Her power of fascination consisted in this abandonment, by which she was enabled to concentrate her whole mind and heart on the character she was depicting.

In this category may be placed many of those cases of hesitation, or stammering, which have been so unmercifully maltreated by surgical operations.

In many of those *impediments*, as they are termed, had the patient been induced, by judicious precept and management, to concentrate the will—to vocalise the breath—the spasmodic striction or apathy of the glottis might have been overcome, and perfect articulation in the end insured, if patience supported the efforts long enough. In this consists the secret of *singing* stammerers; for it is known, though not often *understood*, that those who stammer in their speech are enabled to sing without the slightest hesitation.

Mr. Bishop has very scientifically elucidated this interesting point, in his paper in the last Number of the "Transactions of the Medical Society of London."

It is probable that the remote cause of nervous disorder is the disturbance of that beautiful balance which a healthy system displays. All circumstances which derange the circulation will do this; and therefore we have a chapter on the *Influence of Blood on Nerve.*

In the pathology of the neuroses we daily observe this influence. A plethoric state of the ophthalmic vessels induces intolerantia lucis; while eyes which become weak from overstraining, are usually *bloodshot* from congestion.

These, and similar facts, bear Sir George Lefevre out in his censure of the treatment of all nervous disorders by stimulants and antispasmodics, and in the wide folly of *generalization* in what are termed nervous disorders.

In some persons a periodicity of ailment is often observed. A paroxysm will come on at a certain hour. But this is not so much from a periodical law of nature, as from peculiar condition or employment.

In a languid circulation, the gray, cold dawn will often induce depression, especially if the weak or weary one contemplates difficulties or labour in his diurnal duties. We are told by travellers, that this is sometimes a national trait; in some boreal regions the natives are morose and desponding in the morning, but become gay and cheerful when the day is done. The naturally nervous, as well as the self-sacrificing debauchée, is the slave of a languid or congested circulation in the morning; the latter especially, requiring to be *screwed up*, like Lord Ogleby, for the day, by stimulants.

The blood of the melancholy requires another kind of stimulant—it must be set in motion by cheerful intercourse and sympathising society. Thus in the morning, when the darkness and solitude of night have nourished the brooding of the fiend, all is sombre and gloomy, but the mind gradually brightens toward evening. So Maculloch—"Midnight is the holiday of the nervous patient;" or as another psychologist has it—"La masquerades des nerfs."

All this points to the potent influence of the circulation on the nervous functions—thus ruling over thought, sentiment, opinion, feeling, and expression, with most despotic sway. Even James Boswell, who, though no physiologist, was a close observer of mankind, wrote, fifty years ago, —"The truth is, that we judge of the happiness and misery of life differently at different times, according to the state of our changeable frame. I always remember a remark made to me by a Turkish lady, educated in France—'Ma foi, monsieur, notre bonheur depend de la façon que *notre sang circule.*'"

Then as to repletion—a good (?) dinner in some persons produces a very melancholy train of nervous symptoms. That which makes some, who are endowed with the dura ilia messorum, ('the rigid guts of reapers,' as Byron translates it,) *well,* makes others *ill.* The paroxysms of languor, dulness, and malaise come on about two hours after the meal, when digestion should commence. But the integrity of the vital laws has been disturbed—the gastric acid is, in relation to the bulk of ingesta, in defect or fails in its duty: the mass to be detruded through the pylorus is not the pulpy chyme, but a semi-solid; then ensues a spasm and effete action of the contractile coat of the stomach, and hence one cause of the distressing gastrodynia.

We cannot lay too much stress on the subject of *Nervous Headache* as it is termed: for, we believe this to be often the first degree of that state, the climax of which may be insanity. We cannot, therefore, be too jealous of mal-du-tête, or any uneasiness about the head, although it is often so totally disregarded.

Now, from excess of study, congestion is certain sooner or later, for what is termed cerebral exhaustion, is but another name for venous remora, or congestion. We believe there is no mere neuralgia of the cerebrum. The Creator has wisely ordained its insensibility: for do we not see large slices and masses of brain taken away almost with painless impunity?

It is true, stimulants often relieve this state of congestion, but it is sometimes essential to abstract blood; and the system being relieved of a load, the patient feels *stronger* for the depletion.

If, during violent exercise, we do not perspire—if reaction do not follow cold-bathing, headache and a train of depressing symptoms ensue.

In those systems which may be termed "*used up,*" a very slight exertion will exhaust. With regard to this point, Sir George Lefevre very aptly writes: "Scott exhausted slowly the taper of life; Byron burned out quickly."

In some of our melancholy poets this exhaustion was very strongly evinced. In Cowper, more than once, it induced a suicidal propensity, which led indeed to attempts that were happily not successful.

In Collins, study was at length followed by almost immediate and extreme depression. In the words of Samuel Johnson, who knew him well: "A few minutes exhausted him, so that he was forced to rest upon the couch, till a short cessation restored his powers, and he was again able to talk with his former vigour."

But even the master-mind of Johnson himself was, in early life, "overwhelmed with a horrible hypochondria, with perpetual irritation, fretfulness, and impatience; and with a dejection, gloom, and despair, which made existence misery. Subsequently, he told Mr. Paradise, that he was sometimes so languid and inefficient, that he could not distinguish the hour upon the town-clock."

It is probable, that a little self-control, combined with judicious remedial management, might thwart, at any rate mitigate, many of these morbid influences. The regular use of the shower-bath will often impart most healthful energy to the brain: and a *change* of study will frequently prove salutary: for the brain needs relief from its monotony of thought, even as the stomach requires an occasional change both with regard to diet and medicine. Even two modes of violent exercise may relieve each other. In our early days, we found no *repose* so effectually dissipate the fatigue of dancing, as a hard gallop after the hounds.

In *intermittent* cephalalgia, brow ague, as it is termed, Sir George Lefevre eulogises the use of arsenic and bark. In one case, cinchona and guaiacum combined, acted as a specific.

When these distressing maladies, however, affect the female, the prominent cause is usually uterine sympathy. In these cases, Mistura ferri composita is the panacea.

In the slight headache, heaviness, or obtuseness, which so often attends severe study, a few sighs will often relieve: for the essence of this is congestion from suppressed breathing, by which the blood is both in a state of remora and becomes hypercarbonized.

These oppressive states deep breathing will repeatedly relieve almost as much as exercise, for the salutary effects of exercise do not, of course, depend on mere progression, the swinging of legs and arms, but are the consequences of efficient circulation in insuring the healthy changes in the blood. On this principle, buoyant thoughts will often relieve headache, as surely as melancholy broodings produce it: and this is not a mere metaphysical or mental influence; it is, that pleasure or happiness induces full pulmonary expansion and free circulation; sorrow, on the contrary, suppressed breathing and remora—hence the instinctive act of *sighing* to relieve.

We cannot, however, agree with Sir George Lefevre, when he asserts there is little difference between the exhaustion of debauch and that of study; his expression is, "both are reeling from intoxication!" This is surely rather a licentious figure of speech. Study revives itself in repose, and we usually perfectly recover. There is no liver, or lung, or stomach organically and permanently affected. We may be closer to the mark even when we assimilate two apparent contrasts—excesses of drink and the exhaustion of total abstinence. Regarding the moral beauty of the creed of Father Mathew who can disbelieve? But habit may so induce necessity, that the very breath of life will depend on the continuance of that which would be otherwise a poison and a sin. In the case of a very rigid member of the Wesleyan sect, *teetotalism*, as it is absurdly called, induced so rapid an emaciation and debility, that we found it essential to recommend an instant adoption of his old habits to save his life.

We know, too, the absolute necessity, in many systems, of administering stimuli during our treatment of severe injury. Once when we were taking in at St. Thomas's, one of Barclay's draymen was admitted with severe compound fracture of the tibia and fibula. Observing the prostration and systemic shock, approaching to collapse, we asked the man what had been his usual daily allowance of porter. On his answering from eight to twelve pots per day, we immediately put him on spare diet—six quarts of stout, which probably saved his life, but which would have made very short work in the destruction of a water-bibber.

Like a faithful disciple of Cullen, Sir George Lefevre places CHOLERA and DIABETES among the neuroses. But in the one, spasm is not essential; in the other, affection of the nerves may be called rather the exciting cause than the disorder itself. Even in Asiatic, or, as Cullen would term it, Indian cholera, we have seen rice-water motions pass unconsciously and without cramp. Even the adoption of the term cholera for the Indian malady is, we think, a grand error, as it so widely differs from the English disorder. Perhaps the first impression of the malaria may be on the eighth pair; but if there be a disorder in which the condition of the blood is a point of deep interest, it is this Asiatic flux;—the blood is poisoned and broken up, the serous portion mingled with mucous shreds flowing through the intestinal exhalents, the crassamentum clogging up the vessels in the form of a black pitchy clot. It is, in fact, as we termed it during the former epidemic, *a profuse intestinal diaphoresis.*

The patient dies, as it were, in a state of asphyxiated collapse, unless, if reaction take place, he sinks under the secondary form of the disorder, typhoid fever. Sir George Lefevre regards fever as depending on nervous lesion, thus agreeing with Billing and Copland, and differing with Dr. Stephens, who deemed poisoned blood the essence of fever.

It is argued that nervous influence is the onset of fever, because patients *feel when the fever comes.* Now, if they die of the shock—if, as Armstrong used to say, the *causi morbi* dropped the patient—this was not fever, its phenomena must require blood. It is true that the blood drawn *very early* in fever is not buffed; but if the first symptom be a shiver, the second is a flush; hence there is a conflict, and the triumph of the blood over the nerve.

Regarding the cure of intermittents, much eulogy is passed on the exhibition of three or four doses of calomel, followed up by ten grain doses of sulphate of quinine thrice in a day.

On the subject of atmospheric influence over the neuroses, there are varied opinions. The notion that the wind blowing from certain quarters is unhealthy or distressing, has often been the subject of ridicule, even in the enlightened "Spectator," where a certain *malade imaginaire* was cured of his whim by the nailing of the vane to the westerly point. But this is really no fallacy. There is a quality in the east wind that blows over the cold wet lands of Holland, which is anything but congenial. We ourselves have often felt assured of the prevalence of an east wind, ere we were well awake in the morning, from a peculiar sensation of malaise. It is certain, also, that the pain in a corn portends rain. An electric cloud passing over our heads will sometimes affect us very suddenly. While we were in Paris, a month ago, a sudden and violent gust of wind blew the gates of Notre Dame against us, exactly at midday. In the evening, on our return to Versailles, an invalid relative, whom we were visiting, told us he had not been so well—that about twelve o'clock his symptoms were suddenly aggravated. Was not the disturbance of atmospheric electricity by the storm the source of the paroxysm?

The full discussion of hysteria, hydrophobia, and tetanus, of the vesaniæ, and of demonomania—the illusions of Tasso, Benvenuto Cellini, &c., would be a very tempting field; they are *κατ' εξοχην*, imaginary maladies, nervous disorders; but our limits warn us to forbear.

Many of those disorders thus termed, however, have often, we believe, a real, a physical cause. Still there is many a Mr. Aspen, many a Lady Fanciful, who, as she is assured she looks so well when she is ill, not only aims at convincing her friends, but, in the end, even herself, of her real indisposition.

We know that it is a prevalent fashion among the aristocracy to aim at pallor of the skin; and we fear that vulgar health is too often chased away by the excessive exhibition of calomel. Thus one of two disorders is often induced—either a sort of *ancœmiated* chlorosis, or a reduction of nervous energy, that may produce not only real disease, but a morbid anxiety to be *supposed* to suffer.

We do not feel disposed to enter on the therapeutics of the neuroses, inasmuch as Sir George Lefevre's book refers to so many maladies which cannot be blended with that class. We must, therefore, waive discussion on the superior virtues of digitalis *macerated in œther*, of muriate of ammonia in membranous congestions, of Dr. Malfatti's *aqua lauro cerasi* (prussic acid *drawn mild*, as Sir George Lefevre quaintly terms it) in hysteria, *et hoc genus omne.* And we must merely hint at the potent influence of emotion of the mind in thwarting medicinal treatment; contenting ourselves with a quotation on the subject from quaint old Burton:—

"The body cannot be cured till the mind be satisfied. Socrates, in Plato, would prescribe no physic for Charmides' headache till first he had eased his troublesome mind—body and soul must be cured together, as head and eyes.

"Cœlum non curabis sine toto capite,
Nec caput sine toto corpore,
Nec totum corpus sine animâ."

A word or two, however, on the *prophylaxis* of nervous maladies.

The nerves are valuable servants, but they are desperate and despotic masters. Let them once get the whip-hand, and woe betide their slave. Now, have *we* not an apology to make to *them?* We either coax and pet and indulge them, as we do spoiled children, or work them to the utmost; and then we wonder that their evil qualities turn the tables on ourselves, and render us slaves to the tempter. Now, in policy, as in argument, it is often wise to pit one of our antagonists against another; thus the brunt of the action is drawn off from ourselves. So we believe we might, with a little management, sometimes pit one passion against another; and to this end we have, for the amusement of a languid hour, formed a sort of *Scale of Antagonizing Emotions.* But our limits only allow us a parting glance at those excited conditions of the mind which are so often the spring of nervous maladies, real and imaginary, that is, in civilized life; for, in the nosology of the savage, we should reject some of the adynamiæ and spasmi, and perhaps all the vesaniæ. It is true we cannot always

"Minister to a mind diseased,
Pluck from the memory a rooted sorrow;"

but, by a little self-denial, *mens sana,* to a degree at least, may be preserved to us. If, in the effort, even self-interest or self-gratification be sacrificed, the transient tears of regret will, we may hope, be consecrated, and turned to those of joy and thankfulness when the struggle is over.

Intense impression on the mind is a subject replete with interest. The illusions so often induced by it are contrasted in their influence over the system. They may be consolatory, an agreeable and happy deception, and should, in some cases, be even encouraged. We will glance at a story told by Kotzebue, in illustration. It is of a young lady whose lover died. His harp, on which he was wont to accompany her, hung in her chamber. After a period of melancholy and grief, she touched the chords of her instrument: the harp, *tuned in accordance*, responded. Surprise and terror were at first the consequences; but these now yielded to a romantic melancholy, with a conviction that the spirit of her lover swept the strings of the harp. Her music became her only consolation, until a scientific friend explained to her the principle of phonic harmonies. From that moment the illusion vanished, and she drooped and died. The *nursing* of her illusion might have saved her life.

In other cases the cure of severe malady may be effected on the principle of imparted impetus to the nervous system. This is the *rationale* of the cure of Miss O'Connor of Chelmsford, by Prince Hohenlohe, who was at the moment in Bamberg: of the relief of Miss Fancourt, and of other curious cases, which many would term miraculous.

But if the impression be *foreboding* of misfortune, of course it should be removed if possible. We could cite many cases of those unhappy prognostics, both from dreams and the prophecy or threat of the gipsy, regarding the termination of operations or of parturition. A young lady, Mrs. W., was warned by an offended gipsy to beware of her first confinement. Her mind brooded over the prophecy, and when her child was born, she sunk and died, from no other probable cause. We have notes before us of many other cases.

Now the brain and nervous system of different persons possess very varied degrees of excitability, constituting the endless varieties of temperament and disposition, thus modelling the character, and influencing the actions of mankind. These varieties depend on so many causes and conditions, congenital, hereditary, and casual—so much are they subject to mutual sympathy and reaction, that their discussion would constitute a complete essay on the passions. We can here only offer a transient glimpse of the influence of mind on body.

The first emotion which influences the mind of a child directly it begins to have wishes and hopes, and the consciousness that these may not be fulfilled, is the contrast of its previous tranquillity or content, anxiety—a combination, therefore, of hope and fear; yet the preponderance, from the nature of the mind, being greatly in favour of the former.

Anxiety is prospective sorrow, its subjects various. In that which may be termed moral anxiety, as that of a wife or a mother for the safety of her husband and her child, there is a sacredness which excites our deepest sympathy. Others have a more unholy spring: a heart tainted with pride or avarice, those besetting sins which so deform human nature, and to the pains of which there is no end—for pride and avarice are never satisfied—there is no real meaning, but a negative one, in the word *enough.* These passions are the very bâne of existence. Yet how many, even of those who decry them, cherish the serpents in their bosom, trusting to honour or riches for sublunary happiness, forgetting the monitory lines of Young:

> "Why all this toil for triumphs of an hour?
> What though we wade in wealth, or soar in fame?
> Earth's highest station ends in 'Here he lies;'
> And 'dust to dust' concludes her noblest song."

The feeling of anxiety is one continued heart-ache—it is the dread of something worse than the present. It is progressive in its degree, and therefore more poignant than real sorrow or grief, which is the pain of memory, and which so constantly, from the mere elasticity of the mind, gradually fades and disappears.

For the anxious heart there is often no relief, save from the eloquent lips of sympathetic friendship, or the consolation of religion. If it be not relieved, low nervous fever will be the consequence, with remora of the circulation, inducing local congestions; then, not only are the secretions diminished, but those which are formed are depraved and unhealthy. For so surely as the enlivening passions oxygenize blood, do the depressing emotions accumulate carbon. By this poison a constant morbid and ineffective reaction is going on, which wofully aggravates the original affection. Thus is established a train of nervous maladies—neuralgia, hypochondriasis, melancholy—inducing that corroding action in the brain which, in the words of the Son of Sirach, "consumeth marrow and bone." In the end, if the brain be long oppressed by its poison-blood, tædium vitæ must be the result, the climax of which may be suicide.

During this progress, the system is in a state of universal malaise—all is going wrong. Circulation, digestion, assimilation, nutrition, the nurses of the vital principle, fail; absorption of fat succeeds, and atrophy is the result. In the anxious mother, the secretion of the milk is checked or depraved, by which half-poisonous fluid are the numerous convulsive and gastric diseases of infancy induced.

The influence of anxiety also constantly lights up those latent germs of constitutional disease, which might otherwise never have been developed. The miliary tubercle of phthisis is thus excited to action, and youth and beauty, till then in seemingly blooming health, are at once doomed to decay, and perish.

But the great source of anxiety and its train of ills is to be sought in those ardent longings for worldly possessions, which are the especial debasements of this age. The Satanic passion of pride, which coils like the serpent in almost unconscious folds around the human heart, being the essence of ambition and of avarice, as it is indeed of almost every feeling which disturbs and darkens, and often destroys the life of man.

When this passion is encouraged in youth, it grows with it, and becomes an integral part of existence—the baneful spring of all our actions. It will require, when years have rolled on, an almost superhuman effort for its control— for the metamorphosis or humbling of a soul thus enslaved. Nay, nothing short of the pure light of religion will suffice; a constant leaning on mercy and redemption, and a patient waiting for the fulfilment of the promise.

On the shrine of ambition, man not only sacrifices the nobler sentiments of his soul—his passport to eternal life—but wrecks even his earthly happiness. Even the pride of success soon palls on the sense: the voice of adulation only incites to repeated painful struggles to insure it; and when all earthly grandeur and power are at length attained, the proud and anxious possessor stalks through his painted halls, fumbles his ingots, or his jewels, or his crosses, and then looks forth on his broad lands and frowning forests, and wonders and deplores (that is, if he CAN moralize) that his heart is not sufficiently capacious to enjoy them according to their splendour or their magnitude.

And Mammon, see how he hugs the miser and the gambler to his dark and agitated bosom. Even while he glares on his victims with his frenzied eye, the infatuated monomaniacs do not, or cannot, or will not see the grin of triumph with which he watches his victims to their doom.

We cannot

> "Through the loophole of retreat,
> Look out upon the world,"

agitated as it has been, as it *is* at this moment, with the intense desire of gain, without a thrill of pity and sympathy for the blind votaries of Mammon, who daily and hourly prostrate themselves before the golden image they have themselves set up.

Tranquillity of mind! It were a miracle indeed, if such a condition of brain could be preserved amid the tumult of a stock and share market, in the face of desperate ventures, in which millions may be involved, and families reduced to irretrievable ruin, by the mere dash of the minister's pen; and it were a vain effort to check the headlong course of one on whom the monomania of gaming has taken so deep a hold. Yet while Mammon thus reigns in every alley, the health of the body is sapped, the noble intellect of man is impaired and perverted, the condition of its organ gradually destroyed, the earthly climax of which may be drivelling or raving insanity, involving, alas! that which is of far more awful import—the extreme peril of the immortal spirit.

On the slaves of pleasure, anxiety is ever an attendant demon. True, the orgies of Bacchus and Venus, during their intense excitement, drown the heart and mind in one voluptuous flood, while the cup of nepenthe, or the lips and arms of beauty throw their spell over the senses; but the deep anxiety of after-thought and feeling can never be compensated by a thousand-fold of such enjoyment. And is the penalty merely transient? Alas, it lasts a lifetime! and, if we may contemplate futurity, the scorpion stings of conscience will continue to wound and agonise, when there is even no death, or grave, or hope of pardon, to yield repose to the soul.

It is deeply painful to reflect on the prolific springs of disorder from these slavish passions; the brain and heart are the especial organs into which their poison is infused. Either the intellect or the senses are reduced to a brutal apathy, or the sensitiveness of the nervous system is so morbidly increased, that, on the slightest disappointment, or social competition even, the whole system is deranged, and there is no philosophy, no piety, to tranquillize a mind so subdued, for irreligion must be the dominant principle of such a life. It is almost a jest to write regarding the *health* of creatures so debased—they are all disorder; the tottering and restless gait, the dull and downcast eye, the atrophied or bloated body, the depraved organic functions,—all indicate the ravages that sin has made within.

And, then, the national insanity which has of late, in a sort of Titanic imitative monomania, overspread the globe——But we pause; for this gigantic madness should form the subject of a separate article.

Among those emotions which, in contrast, are from the first asthenic, we must only allude to grief and its prototypes, as one fertile source of deep or protracted nervous maladies. The intense degree of grief is all-absorbing. The mind broods over the one subject of its woe, and so reluctant is it to admit another, that it is often annoyed by conversation of friends, or even impression on the senses. Hence the deep mourner retires into lonely seclusion; and soon may be lighted up a train of feelings as distressing as they are obnoxious to remedy—melancholy. When this sad condition is the result of moral causes, time must, of course, be given; but the *maladie imaginaire* is often the result of mere corporeal derangement. This we may so far set right; but even then how often do we leave the work half done, and, in disregard of our moral principles, let judgment go by default.

Society and sympathy, *wisely managed,* will be life itself to the sensitive heart; without them, it will droop and decay. The savage may roam in the desert uninfluenced by its desolation—he is familiar with solitude, he makes it a world of his own; but the cell of a social man is peopled too often, not by congenial spirits, but by spectres that fright the soul from its propriety.

And now may we conclude our remarks on the pathology of nervous maladies, the symptoms, causes, and treatment of which we may learn from other books, or in the schools, by merely glancing at those principles on which *prophylaxis* so much depends.

If we believe in the irritation or disturbance of mind as a fertile source of the neuroses, we may also believe that the induction of a contrasted state of mind would prove a curative or preventive. This condition would be that which is the antipodes of pride, envy, hatred, and low ambition (which, as Lord Bacon writes, "have no holidays")—that which we term repose—contentment, tranquillity, *happiness.* By mental repose we do not mean the apathetic state of the thoughtless or the slothful; the *dolcè far niente* of the useless do-nothing is the mere scum on the surface of the cup of idleness, which contains a poisonous bitter in its dregs. Under the placid condition of mind, not only is the *vis medicatrix* allowed to exert its potent influence, but the various functions of the body are almost ensured or restored to their former integrity: "To laugh and grow fat" has become a proverb.

Yet to insure this happy mood how multiform are the precepts—amusements and moderate occupation, and those most congenial to the disposition. But this mental election must not be negative; the mind must be brought, not only to forego those perilous pleasures of sense and of sensibility to which luxury and sloth are so naturally prone, but also to *act on* the subject of its thoughts, not with fatigue and labour, but with that degree of energy which will afford food for immediate reflection, and the memory of which will be the constant spring of tranquil satisfaction. Thus, as Burke has enjoined, "we should live pleasant." To ensure this, requires often a high degree of self-control, as well as the sympathy of friendship. The greatest caution in conversation is sometimes essential; allusions to subjects which are agreeable, congenial, and consolatory to the invalid, should be adopted, both in conversation and in reading; and objects of beauty and interest should as much as possible be presented to the mind; for it has been observed how influential are odour, and colour, and form, in mitigation of more decided maladies.

The philosophic mind will often be successful in controlling and preserving a tranquil temper; but, under suffering, even philosophy may fail, if uncombined with true and practical religion, in which the Christian and cardinal virtues are conspicuous. Benevolence, charity—indeed, *any act by which benefit is conferred on mankind from pure and worthy motives,* must succeed in inducing that happy mood which confers on the heart and mind, contentment—which sheds the poppy and the balm over the pillow of health, and constitutes often half the remedy of disease.

But after all, is this lesson so easy in this excited, scientific, artificial age, in which we have wandered so far from our primitive simplicity? We have, in truth, so multiplied our wants, that we become restless if we do not accomplish all the mind can conceive: like Ariel, we would put a girdle round the earth in forty minutes. In short, we must have all that is in *posse* to be in *esse.*

Then, have not empiricism, and book-making, and the vaunting of specifics, magnified our imaginary maladies to excess? Dyspepsia has not certainly diminished, although so many learned tracts, popular and scientific, have been scribbled upon it; nor with all our essays on thoracic pathology, combined even with our moral tracts, have we banished valvular disease or heartache from the breasts of the lieges. Have we, in short, added to our moral happiness by this gigantic march of science? But we forget we are writing for Paradise, and not for earth: and, however we may hope, we shall never be able, with or without the parallelograms of Robert Owen, to connect the state of our restless world with that of Utopia.

